# Association with emotional information alters subsequent processing of neutral faces

**DOI:** 10.3389/fnhum.2014.01001

**Published:** 2014-12-18

**Authors:** Lily Riggs, Takako Fujioka, Jessica Chan, Douglas A. McQuiggan, Adam K. Anderson, Jennifer D. Ryan

**Affiliations:** ^1^Rotman Research InstituteToronto, ON, Canada; ^2^Department of Psychology, University of TorontoToronto, ON, Canada; ^3^Department of Psychiatry, University of TorontoToronto, ON, Canada

**Keywords:** emotion, magnetoencephalography (MEG), neuroimaging, eye movement monitoring, faces

## Abstract

The processing of emotional as compared to neutral information is associated with different patterns in eye movement and neural activity. However, the ‘emotionality’ of a stimulus can be conveyed not only by its physical properties, but also by the information that is presented with it. There is very limited work examining the how emotional information may influence the immediate perceptual processing of otherwise neutral information. We examined how presenting an emotion label for a neutral face may influence subsequent processing by using eye movement monitoring (EMM) and magnetoencephalography (MEG) simultaneously. Participants viewed a series of faces with neutral expressions. Each face was followed by a unique negative or neutral sentence to describe that person, and then the same face was presented in isolation again. Viewing of faces paired with a negative sentence was associated with increased early viewing of the eye region and increased neural activity between 600 and 1200 ms in emotion processing regions such as the cingulate, medial prefrontal cortex, and amygdala, as well as posterior regions such as the precuneus and occipital cortex. Viewing of faces paired with a neutral sentence was associated with increased activity in the parahippocampal gyrus during the same time window. By monitoring behavior and neural activity within the same paradigm, these findings demonstrate that emotional information alters subsequent visual scanning and the neural systems that are presumably invoked to maintain a representation of the neutral information along with its emotional details.

## INTRODUCTION

We are constantly involved in the interpretation of social and emotional cues from those around us. Such cues can be conveyed via facial expressions (Adolphs, 2003) and facial appearance (Bar et al., 2006; Willis and Todorov, 2006), as well as by biographical information (Todorov and Uleman, 2002, 2003, 2004). For example, imagine being at a party and seeing two different people who appear neutral and non-threatening. However, right before you meet them, you are told that one person has just gotten out of jail for murder and the other person is working on a Ph.D. Research from social psychology suggests that from this minimal information, you will then form a rapid, perhaps automatic, and very different impression of the two people (Todorov and Uleman, 2002, 2003, 2004). As a consequence of forming such rapid impressions, differences may occur in the way in which we perceive otherwise neutral information (i.e., the person’s face).

There are now a number of studies showing that the processing of emotional versus neutral stimuli is characterized by different patterns of eye movement behavior (e.g., Calvo and Lang, 2004, 2005; Nummenmaa et al., 2006; Riggs et al., 2010, 2011). However, the manner in which we perceive information is driven not only by stimulus-bound characteristics, but also by prior knowledge (for review see: Hannula et al., 2010). In a classic study by Loftus and Mackworth (1978), the researchers showed that prior semantic knowledge altered the way participants scanned scenes within the first few fixations. Given the influence of prior knowledge on viewing neutral stimuli, it is possible that emotional information may also influence subsequent viewing of neutral stimuli.

The goal of the present study was to examine whether presenting emotional information would influence subsequent viewing patterns and underlying neural activity of neutral faces, when this may occur and in what manner. Specifically, does presenting emotional information ‘prime’ us to actually perceive the neutral stimulus in a different way, and/or does it modulate post-perceptual processes such as elaboration and binding? Previous neuroimaging studies suggest that while perceptual processing occurs largely within the first 250 ms after stimulus onset in posterior sensory cortices, conceptual/semantic processes, and/or the retrieval of associated information are largely purported to occur during later time windows in frontal and medial temporal regions (e.g., 500–1500 ms; Donaldson and Rugg, 1999; Schweinberger et al., 2002). Therefore, in order to determine how and when emotion may influence visual processing of neutral information, it is important to use a neuroimaging technique with very fine temporal resolution such as magnetoencephalography (MEG).

Magnetoencephalography is a non-invasive neuroimaging technique that measures the magnetic field differences produced by population of neurons (Hämäläinen et al., 1993; Hari et al., 2000), providing recording of neural activity with temporal resolution on the order of milliseconds and with good spatial resolution (Miller et al., 2007). Critically, through its precise timing information, MEG has been successfully utilized to study emotion processing (Cornwell et al., 2008; Hung et al., 2010), as well as how knowledge and/or prior experience can influence the manner by which visual processing occurs (Ryan et al., 2008; Riggs et al., 2009). In a MEG study by Morel et al. (2012), the researchers examined whether prior association with auditorily presented negative, positive, or neutral information changed the manner by which neutral faces are retrieved after a delay (1–7 min). The authors found that neural responses in the bilateral occipital–temporal and right anterior medial temporal regions differentiated neutral faces previously paired with positive versus negative and neutral information as early as 30–60 ms after face onset. In other words, prior association with positive information modulated the earliest stages of perceptual processing during retrieval. A similar ERP by Smith et al. (2004) reported differences over the lateral temporal and frontal–temporal regions during retrieval of neutral items studied in an emotional (positive and negative) versus neutral context, albeit during a later time frame (300–1900 ms). However, it remains unclear whether emotional information has an immediate influence on the online visual processing of neutral information, prior to any contribution from memory consolidation (as may have occurred in Morel et al., 2012).

In the present study, participants were presented with a neutral face (Face 1) followed by either a negative or a neutral sentence about that person, and then the neutral face was presented again in isolation (Face 2) ^1^. In order to examine whether the assignment of an emotional label to a neutral face would influence processing, it was critical to present them separately. Otherwise, it would be impossible to disentangle the effects of processing a neutral item that has been presented with the emotional information from the effects of processing the emotional stimulus itself. Visual scanning and neural activity were recorded simultaneously using eye movement monitoring (EMM) and MEG, respectively. This allowed us to directly link emotion-modulated differences in behavior with neural response. Herdman and Ryan (2007) first demonstrated the feasibility of recording eye movement behavior and neural activity simultaneously within a single paradigm; here, this unique combination of technologies was applied to the study of emotion. It was predicted that if emotion influenced visual processing of subsequent neutral information, then differences in eye movement patterns and neural activity should occur during viewing of Face 2. Specifically, viewing of faces presented after a negative versus neutral sentence should result in increased viewing to the internal features of the face (Calder et al., 2000; Green et al., 2003), especially the eye region (Adolphs et al., 2005; Itier and Batty, 2009; Gamer and Buchel, 2009). It should also elicit enhanced activation of regions implicated in emotion processing such as the amygdala, cingulate, and anterior insula (Adolphs, 2002; Haxby et al., 2002; Phelps, 2006; Chen et al., 2009), attention and facial processing such as the precuneus and fusiform gyrus (Cavanna and Trimble, 2006; Deffke et al., 2007), item binding such as the prefrontal cortices and medial temporal lobe (Cohen et al., 1999; Badgaiyan et al., 2002), and the processing of person identity and biographical information such as the superior temporal sulcus (STS; Haxby et al., 2002; Todorov et al., 2007). Further, if emotion changes perceptual processing of neutral information, then such eye movement and neural differences should manifest early during viewing (i.e., within the first fixation and <250 ms in the brain). However, if emotion influences post-perceptual processes such as retrieval and semantic/conceptual processing, then eye movement and neural differences should manifest later during viewing (i.e., after the first fixation and >500 ms in the brain). If differences are found between processing neutral faces presented following a negative versus neutral information, this would suggest that emotion can influence the visual processing, and the mental representation of subsequent neutral stimuli in a top–down manner that is independent of its physical properties. Thus, we may not only form different impressions of the ‘murderer’ versus ‘student,’ but the very manner in which we process those faces may also be different.

## MATERIALS AND METHODS

### PARTICIPANTS

Twelve young adults (mean age = 21.6 years, SEM = 0.71, 5 males) from the Rotman Research Volunteer Pool participated for $10 per hour. All participants had no history of neurological or clinical disorders, no history of head trauma and had normal or corrected-to-normal vision. All participants were either native English speakers or had attended school in English for at least 12 years. All participants provided informed written consent by the guidelines of the Baycrest Hospital Research Ethics Board prior to the start of the experiment.

### STIMULI AND DESIGN

The stimuli used consisted of 300 black and white, non-famous male faces with a neutral expression selected from a database of face images as outlined by Schmitz et al. (2009). Briefly, the photographs showed front-view non-expressive faces. Faces were cropped and did not contain hair or other non-facial features. To prevent discrepancies in the spatial orientation and location of the face stimuli over trials, the eyes and philtrum of each image were aligned to a standard 3-point Cartesian space with the nose at the center. Similar correction procedures were also used to equate low-level visual perceptual differences such as luminance and shading (for more details see: Schmitz et al., 2009). The faces were placed against a uniform black background – the resulting image was 300 × 300 pixels. Each face was randomly paired with a negative (e.g., “This person is a rapist”) and a corresponding neutral sentence (e.g., “This person is a linguist”). Each pair of sentences differed only by one critical word, which was either negative or neutral, and was matched for the number of syllables. All of the sentences began with “This person…” in order to encourage participants to process the face and sentence as a pair. All of the sentences were previously rated by 10 participants (mean age = 21.3, SEM = 0.96, 4 males) on a 5-point Likert scale on dimensions of familiarity, coherence, tabooness, valence, and arousal. Negative and neutral sentences were matched for familiarity [*t*(99) = -1.30, *p* = 0.20] and coherence [*t*(99) = -0.03, *p* = 0.97]. Critically, negative sentences were judged to be more taboo [*t*(99) = 26.13, *p* < 0.0001], negative [*t*(99) = -25.12, *p* < 0.0001], and arousing [*t*(99) = 24.24, *p* < 0.0001]. The mean values can be found in **Table 1**.

**Table 1:** The mean and SEM of ratings for the negative and neutral sentences used in experiment.

	Mean for negative sentences (SEM)	Mean for neutral sentences (SEM)
Familiarity	4.74 (0.03)	4.79 (0.03)
Coherence	4.84 (0.02)	4.84 (0.02)
Tabooness	3.09 (0.08)	1.06 (0.02)
Valence	1.75 (0.04)	3.20 (0.04)
Arousal	2.68 (0.06)	1.21 (0.02)

Participants were shown 200 unique faces across five blocks (40 per block). Each face was presented with either a negative (100) or neutral (100) sentence. Faces were displayed in a pseudo random order such that no more than three negative or three neutral face-sentence-face pairings appeared in succession. Each study block contained 20 faces paired with a negative sentence, and 20 faces paired with a neutral sentence. Counterbalancing was complete such that each face appeared with a neutral and negative sentence equally often across participants.

### PROCEDURE

**FIGURE 1:**
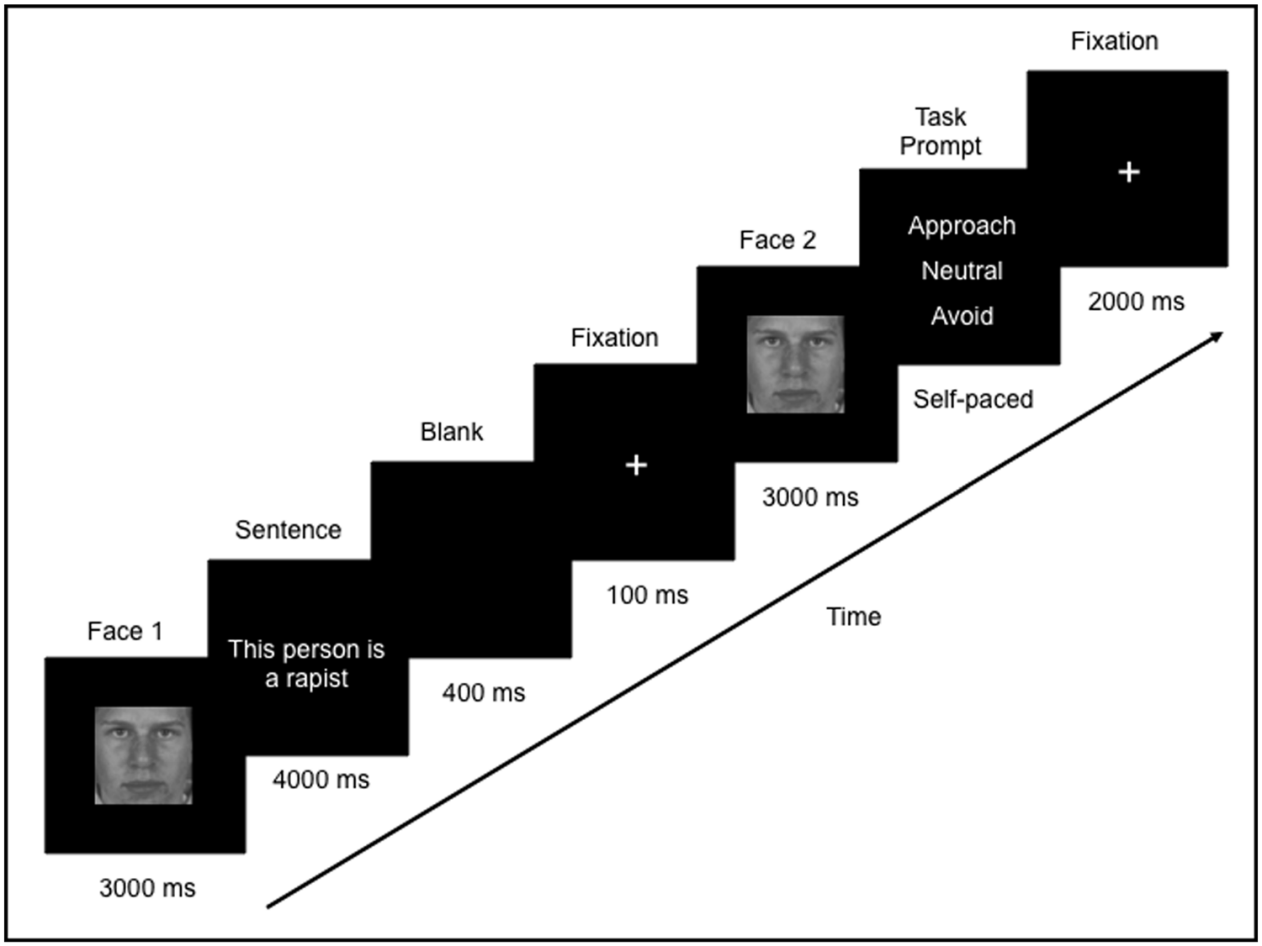
**Example of one trial in the study.** Participants freely viewed a face (Face 1) followed by a sentence that was either negative or neutral. The same face was presented again (Face 2) and participants were asked to judge whether they would want to approach, avoid or neither approach or avoid (remain “neutral” to) that person.

Eye movements and neural activity were recorded simultaneously throughout the experiment. Participants were told that they would see a face, followed by a sentence that describes that person, and then they would see the face again. During each trial, a face was presented for 3000 ms (Face 1), followed by a sentence which was presented for 4000 ms. Since the sentences varied in length, a blank screen (400 ms) followed by a fixation cross (100 ms) was presented in order to direct the participants’ eyes back to the center of the screen. Following this, Face 1 was re-presented again, in isolation, for 3000 ms (Face 2; **Figure 1**). Participants were then asked to indicate via a button press whether they would want to approach, avoid, or stay neutral to the person. The purpose of the task was to encourage participants to process the meaning of the face-sentence pairings. Throughout the trial, participants were instructed to freely view the faces and sentences presented. There was a 2000 ms inter-trial interval, which consisted of a fixation cross in the center of a blank screen. Participants were instructed to fixate on the central cross whenever it appeared. In this way, participants began each trial fixated on the center of the screen (i.e., over the nose region of the face).

### DATA ACQUISITION

Eye movements were measured with a SR Research Ltd. Eyelink 1000 remote eyetracker. This recorded eye movements at a rate of 500 Hz and with a spatial resolution of 0.1°. A 9-point calibration was performed at the start of each block followed by a 9-point calibration accuracy test. Calibration was repeated if the error at any point was more than 1°. Drift corrections were performed at the beginning of each trial if necessary. Poorly calibrated trials were deleted from the eye movement analyses; this resulted in a total of nine deleted trials across all participants.

Magnetoencephalography recordings were performed in a magnetically shielded room, using a 151-channel whole head first order gradiometer system (VSM-Med Tech Inc., Port-Coquitlam, BC, Canada) with detection coils uniformly spaced 31 mm apart on a helmet-shaped array. Participants sat in an upright position, and viewed the stimuli on a back projection screen that subtended approximately 31° of visual angle when seated 30 inches from the screen. The MEG collection included the signal of the stimulus onset by recording the luminance change of the screen. Participant’s head position within the MEG was determined at the start and end of each recording block using indicator coils placed on nasion and bilateral preauricular points. These three fiducial points established a head-based Cartesian coordinate system for representation of the MEG data. In order to specify/constrain the sources of activation as measured by MEG and to co-register the brain activity with the individual anatomy, a structural T1 MRI was also obtained for each participant using standard clinical procedures with a 3T MRI system (Siemens Magnetom Trio whole-body scanner) located at Baycrest Hospital.

### EYE MOVEMENT ANALYSIS FOR FACES

Differences in the eye movement patterns made to faces that had been paired with negative versus neutral sentences were taken as evidence that the processing of faces may be changed via the emotional information that had preceded them. Therefore, we compared eye movement behavior during viewing of Face 2 following a negative sentence (Face2-Negative) versus that of Face 2 following a neutral sentence (Face2-Neutral). As a control condition, we also compared eye movement during viewing of Face 1 that preceded a negative (Face1-Negative) versus neutral sentence (Face1-Neutral). Since these faces had not yet been paired with either a negative or a neutral sentence, no differences were expected in measures of eye movement behavior. Viewing to regions corresponding to the location of features within the face, i.e., eyes, nose, and mouth, were examined.

Analyses were conducted for eye movement measures of early viewing and measures of overall viewing, that is, viewing behavior that occurs throughout stimulus presentation (for more details see: Hannula et al., 2010). Measures of early viewing included: the start time of the first fixation, duration of the first gaze, and number of fixations within the first gaze. A fixation was defined as the absence of any saccade (e.g., the velocity of two successive eye movement samples exceeds 22° per second over a distance of 0.1°) or blink (e.g., pupil is missing for three or more samples) activity. Start time of the first fixation is the time, from stimulus onset, at which a fixation was first directed to a particular region of interest. Duration of the first gaze is the total time spent during the first instance the viewer’s eye movements enter a particular region, prior to leaving it. Number of fixations within first gaze is the total number of fixations during the first instance the eyes enter a particular region before moving out of that region. Measures of overall viewing included: average duration of all eye fixations, number of fixations and number of transitions into a region of interest.

### MEG ANALYSIS FOR FACE PROCESSING

Similar to the eye movement analysis for faces, spatiotemporal differences in neural activity underlying viewing of faces that had been paired with negative versus neutral sentences were taken as evidence that the processing of faces may be changed via emotional details that had preceded them. We compared neural activity during viewing of Face2-Negative versus Face2-Neutral. A comparison of neural activity underlying viewing of Face1-Negative versus Face1-Neutral was also included as a control condition.

Source activity was estimated using the synthetic aperture magnetometry (SAM) minimum-variance beamformer (Van Veen et al., 1997; Robinson and Vrba, 1999) across the whole brain on a grid with regular spacing of 8 mm. The beamformer analysis, using the algorithm as implemented in the VSM software package, was based on individual multisphere models, for which single spheres were locally approximated for each of the 151 MEG sensors to the shape of the cortical surface as extracted from the MRI (Huang et al., 1999). The SAM beamformer minimizes the sensitivity for interfering sources as identified by analysis of covariance in the multichannel magnetic field signal while maintaining constant sensitivity for the source location of interest. The covariances were calculated based on the entire trial duration (-1000 ms to 10,500 ms) with low-pass filter at 30 Hz. Thereafter, the resultant SAM weights were applied to the MEG sensor data separately for each epoch of interest based on an event-related spatial-filtering approach (ER-SAM; Robinson, 2004; Cheyne et al., 2006) as used in our previous studies (Moses et al., 2009; Fujioka et al., 2010). Using the MEG data of the entire segment of the trial to compute SAM separately for the epoch of interest is necessary because as mentioned above, the spatial filter is dependent on the covariance of the MEG measures. Therefore, this procedure ensures that the resultant ER-SAM source activities for each condition reflect differences in the brain activity patterns and not to differences in the spatial filters computed from different epochs of interest. Further, an advantage of using ER-SAM is that it removes differences between the experimental conditions that are not time-locked, for example, potential eye movement differences. Therefore, observed differences in neural activity reflect differences in cognitive processing that are time-locked to the onset of the neutral face.

The MEG data were epoched from 100 ms prior to stimulus onset to 2800 ms after stimulus onset for each condition (i.e., Face2-Negative, Face2-Neutral). Before applying the beamformer to each single epoch of magnetic field data, artifact rejection using a principal component analysis (PCA) was performed such that field components larger than 1.5 pT at any time were subtracted from the data at each epoch (Kobayashi and Kuriki, 1999). This procedure is effective in removing artifacts with amplitudes significantly larger than brain responses, such as those caused by eye blinks and saccades (Lins et al., 1993; Lagerlund et al., 1997). The PCA approach has been used extensively in both EEG and MEG studies as a means of identifying and removing eye movement artefact (e.g., Riggs et al., 2009; Ross and Tremblay, 2009; Fujioka et al., 2014). By low-pass filtering the MEG data at 30 Hz, we also reduced the influence of micro-saccades, which are low amplitude but occur at high frequency bands (>30 Hz, Carl et al., 2012).

Using the spatial filter, single-epoch source activity was first estimated as a pseudo-Z statistic for each participant and each condition. The time series of the source power within 0–30 Hz was then calculated for each single source waveform. Finally, the representation of the evoked response was obtained as a time series of the average power across trials normalized to the pooled variance across the duration of trials for each voxel. These individual functional maps were then spatially transformed to the standard Talairach space using AFNI (National Institute of Mental Health, Bethesda, MD, USA) and averaged across all participants. For each participant, functional data from the MEG was co-registered with their structural MRIs by using the same fiducial points defined by three indicator coils placed on the nasion and bilateral periauricular points.

In order to capture the influence of emotion on early perceptual processing of neutral faces, we examined MEG data (0–250 ms) with sliding time windows of 100 ms, i.e., 0–100, 50–150, 100–200, and 150–250 (e.g., Walla et al., 2001). In order to capture the influence of emotion on both early and later processing, we also examined MEG data (0–1200 ms) with longer sliding time windows of 600 ms (i.e., 0–600, 300–900, 600–1200 ms; Moses et al., 2009; Fujioka et al., 2010). Spatiotemporal differences in the brain responses to viewing faces presented after negative versus neutral sentences were characterized using the partial least squares (PLS) multivariate approach (McIntosh and Lobaugh, 2004). The PLS approach has been successfully used for time-series neuroimaging data in MEG (Moses et al., 2009; Fujioka et al., 2010). Critically, PLS examines all voxels in the brain at once, thereby circumventing the need to correct for multiple comparisons (e.g., McIntosh et al., 2004). In order to accommodate computation demands, the Talairach-transformed individual functional maps for each participant were down-sampled to 78 Hz, which resulted in volumetric maps every 12.8 ms, and used as input for a mean-centered PLS analysis. Mean centering allowed values for the different conditions to be expressed relative to the overall mean. Using this type of analysis, activation patterns that are unique to a specific condition will be emphasized whereas activations that are consistent across all conditions, such as primary visual activation, will be diminished.

The input of PLS is a cross-block covariance matrix, which is obtained by multiplying the design matrix (an orthonormal set of vectors defining the degrees of freedom in the experimental conditions), and the data matrix (time series of brain activity at each location as columns and subjects within each experimental condition as rows). The output of PLS is a set of latent variables (LVs), obtained by singular value decomposition applied to the input matrix. Similar to eigenvectors in PCA, LVs account for the covariance of the matrix in decreasing order of magnitude determined by singular values. Each LV explains a certain pattern of experimental conditions (design score) as expressed by a cohesive spatial–temporal pattern of brain activity. The significance of each LV was determined by a permutation test using 500 permuted data with conditions randomly reassigned for recomputation of PLS. This yielded the empirical probability for the permuted singular values exceeding the originally observed singular values. An LV was considered to be significant at *p* ≤ 0.05. For each significant LV, the reliability of the corresponding eigen-image of brain activity was assessed by bootstrap estimation using 250 resampled data with subjects randomly replaced for recomputation of PLS, at each time point at each location. The bootstrap method is a measure of reliability across participants and ensures that the results are not being driven by only a few participants. A high bootstrap value indicates that an effect is observed across most, if not all, of the participants. Sources that were spatially distinct with a bootstrap ratio of ±3 at the local maxima were examined.

## RESULTS

Participants responded “avoid” to 78.4% of faces which had been paired with negative sentences, and “neutral” or “approach” to 86.5% of faces which had been paired with neutral sentences. This suggests that faces presented with negative sentences were primarily interpreted as negative, whereas faces presented with neutral sentences were not interpreted as negative. In the current study, all of the faces were included in the eye movement and MEG analysis in order to increase signal-to-noise ratio.

### VIEWING OF FACES

Analyses of variance (ANOVA) were conducted on measures of viewing to Face 2 using emotion (negative, neutral) and face feature (eyes, mouth, nose) as within-subject factors. As a control, the same analyses were also conducted for viewing to Face 1 and no significant effects were found (all *p*’s > 0.1), therefore, for brevity, we only describe viewing to Face 2. Mean values are found in **Table 2**.

**Table 2:** The mean and SEM for early **(A)** and overall **(B)** measures of viewing to different features within Face 1 and Face 2.

	Face 1		Face 2	
	Negative (SEM)	Neutral (SEM)	Negative (SEM)	Neutral (SEM)



**(A) Early measures of viewing:**
**Start time of first fixation (ms)**
Eyes	509.33 (52.22)	507.57 (49.73)	409.94 (58.87)	375.85 (65.96)
Nose	113.62 (19.23)	129.13 (15.95)	313.92 (100.25)	393.88 (89.70)
Mouth	1323.13 (151.85)	1322.94 (160.66)	1474.30 (95.47)	1417.68 (77.32)
**Duration of first gaze (ms)**
Eyes	1104.0 (133.5)	1088.6 (122.4)	1035.4 (115.3)	927.0 (86.9)
Nose	404.3 (55.5)	422.6 (66.3)	471.4 (80.0)	453.9 (74.9)
Mouth	275.6 (16.6)	329.4 (37.5)	324.7 (29.5)	323.7 (29.9)
**Number of fixations within first gaze (#)**
Eyes	0.41 (0.04)	0.41 (0.04)	0.39 (0.04)	0.35 (0.03)
Nose	0.17 (0.02)	0.17 (0.02)	0.19 (0.03)	0.18 (0.02)
Mouth	0.11 (0.01)	0.12 (0.01)	0.12 (0.00)	0.11 (0.01)



**(B) Overall measures of viewing:**
**Average duration of fixations**
Eyes	332.07 (38.33)	333.59 (40.07)	325.42 (24.12)	316.45 (26.68)
Nose	300.76 (25.28)	309.47 (32.28)	319.95 (31.18)	316.06 (29.84)
Mouth	265.91 (14.90)	304.07 (39.40)	292.63 (24.09)	287.55 (21.39)
**Number of fixations**
Eyes	5.60 (0.48)	5.63 (0.45)	5.41 (0.50)	5.53 (0.49)
Nose	2.38 (0.27)	2.35 (0.27)	2.26 (0.29)	2.33 (0.30)
Mouth	0.27 (0.05)	0.28 (0.05)	0.27 (0.06)	0.30 (0.01)
**Number of transitions**
Eyes	1.82 (1.00)	1.81 (0.09)	1.77 (0.10)	1.93 (0.09)
Nose	1.75 (0.13)	1.72 (0.12)	1.56 (0.14)	1.63 (0.14)
Mouth	0.25 (0.05)	0.25 (0.05)	0.24 (0.05)	0.26 (0.06)

Of note, the very first fixation was typically in the nose region because this was at the center of the screen, or outside of the pre-specified ROIs because participants had not returned their gaze to the center of the screen yet. Participants spent significantly more time [*F*(1,11) = 4.76, *p* = 0.05, $\eta_{p}^{2}$ = 0.30] and directed marginally more fixations [*F*(1,11) = 3.62, *p* = 0.08, $\eta_{p}^{2}$ = 0.25] during the first gaze to the different features of faces paired with negative versus neutral sentences. There was also a marginal interaction between emotion and face feature for the duration of the first gaze [*F*(2,22) = 3.10, *p* = 0.07, $\eta_{p}^{2}$ = 0.22] and the number of fixations within first gaze [*F*(1,11) = 2.92, *p* = 0.08, $\eta_{p}^{2}$ = 0.21]. Follow-up *t*-tests revealed that during the first gaze, participants spent significantly more time [*t*(11) = 2.31, *p* < 0.05] and directly marginally more fixations [*t*(11) = 1.93, *p* = 0.08] to the eye region of faces paired with negative versus neutral sentences, but not for other regions of the face (*p*’s > 0.1). There were no significant effects of emotion for the start time of the first fixation (*p*’s > 0.1). Across both types of faces, the first fixation to the eye region occurred at around 400 ms after stimulus onset. Thus, emotion-modulated viewing differences found during the first gaze to the eye region occurred approximately between 400 and 1400 ms (the duration of the first gaze was approximately 1000 ms, see **Table 2**).

Viewing across the entire trial revealed fewer fixations [*F*(1,11) = 4.34, *p* = 0.06, $\eta_{p}^{2}$ = 0.28] and fewer transitions between face features [*F*(1,11) = 10.51, *p* < 0.01, $\eta_{p}^{2}$ = 0.49] for faces paired with negative versus neutral sentences. A significant interaction for the number of transitions [*F*(2,22) = 5.40, *p* < 0.05, $\eta_{p}^{2}$ = 0.33] revealed that participants made fewer transitions into the eye region of faces paired with negative versus neutral sentences [*t*(11) = -3.45, *p* < 0.01], whereas there was no difference in viewing of the other features (*p*’s > 0.1). No significant effects were found for the measure of average fixation duration (*p*’s > 0.1).

In summary, emotion led to early changes in the subsequent viewing of neutral faces. Specifically, negative versus neutral sentences led to an initial increase in viewing of the eye region of neutral faces, and perhaps as a consequence, decreased overall sampling (i.e., fewer fixations and transitions) across the remainder of the trial.

### NEURAL ACTIVITY TO FACES

**FIGURE 2:**
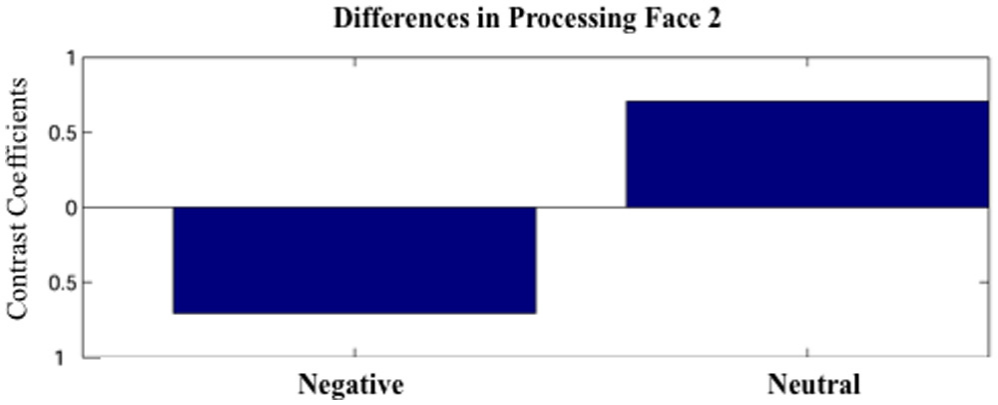
**The first latent variable (LV1) obtained by the partial least squares (PLS) analysis.** The LV1 contrast coefficient revealed that processing faces paired with negative versus neutral sentences yielded unique spatio-temporal patterns of brain activation during 600–1200 ms after face onset.

Partial least squares analysis did not reveal any differences between Face1-Negative and Face1-Neutral. For Face 2, PLS analysis did not yield any significant design LVs for the short 100 ms time windows (*p*’s > 0.05). However, one significant design LV (LV1) emerged for the time window 600–1200 (*p* < 0.05; **Figure 2**).

**FIGURE 3:**
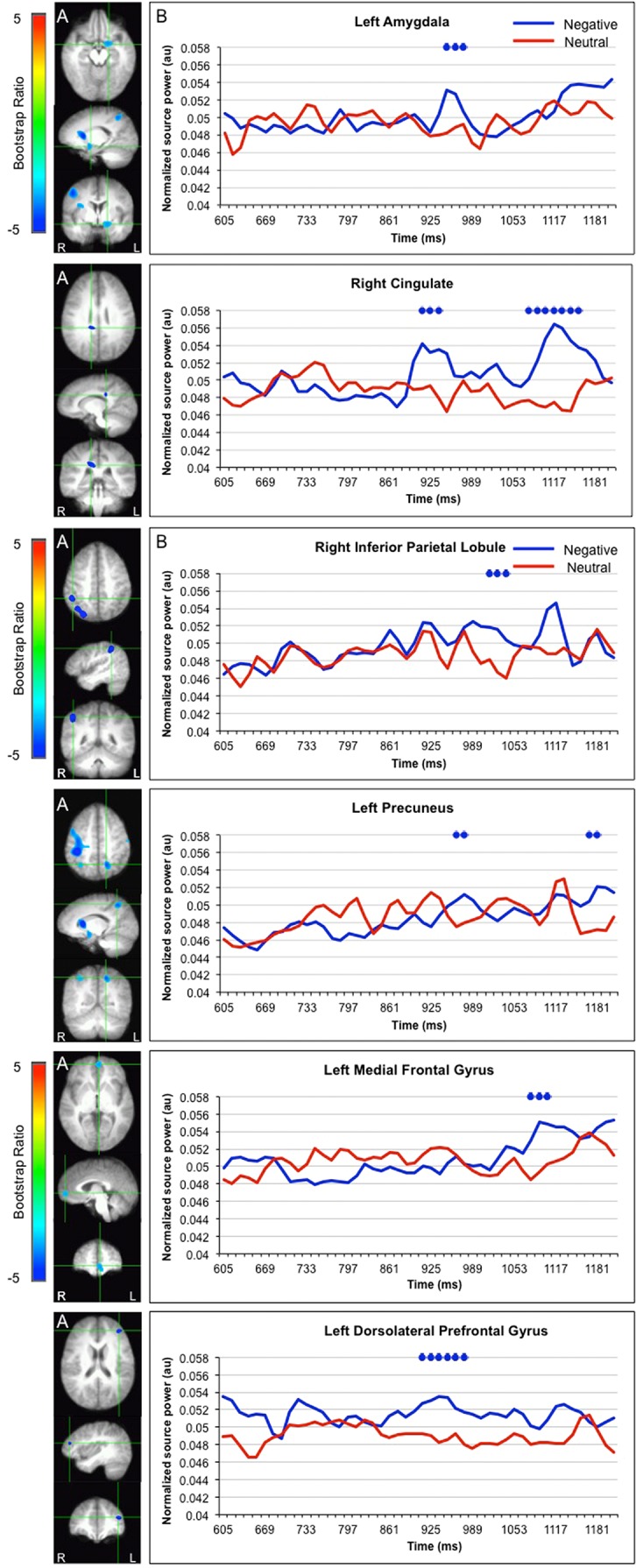
** Sources showing stronger activation for faces paired with negative as compared to neutral sentences. (A)** The brain areas associated with the PLS bootstrap ratio less than –3 in the LV1. **(B)** Corresponding ER-SAM waveforms from sources identified in PLS bootstrap ratio maps. Blue dots denote bootstrap ratios <–3, and red dots denote bootstrap ratios >3. Since no significant differences were found for 0–600 ms, the x-axis begins at around 600 ms in order to fully visualize the emotion-modulated differences observed.

LV1 revealed greater activation for faces paired with negative versus neutral sentences in emotion processing regions such as the left amygdala (950–976 ms), right cingulate (925–938, 1091–1155 ms), and left medial frontal gyrus (1078–1117 ms), in posterior regions such as the left precuneus (989–1053 ms) and right inferior parietal lobule (1014–1040), and in the dorsolateral prefrontal cortex (912–976 ms; **Figure 3**). These areas were represented by the local maxima/minima of the bootstrap ratio of the LV1.

**FIGURE 4:**
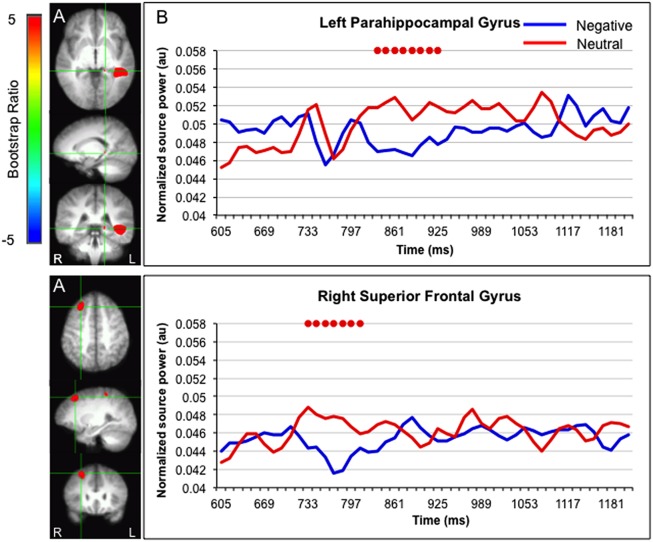
**Sources showing stronger activation for faces paired with neutral as compared to negative sentences. (A)** The brain areas associated with the PLS bootstrap ratio more than 3 in the LV1. **(B)** Corresponding ER-SAM waveforms from sources identified in PLS bootstrap ratio maps. Red dots denote bootstrap ratios >3. Since no significant differences were found for 0–600 ms, the x-axis begin at around 600 ms in order to fully visualize the emotion-modulated differences observed.

Greater activation for faces paired with neutral versus negative sentences was found in the left parahippocampal gyrus (835–925 ms) and right superior frontal gyrus (733–810 ms; **Figure 4**).

**FIGURE 5:**
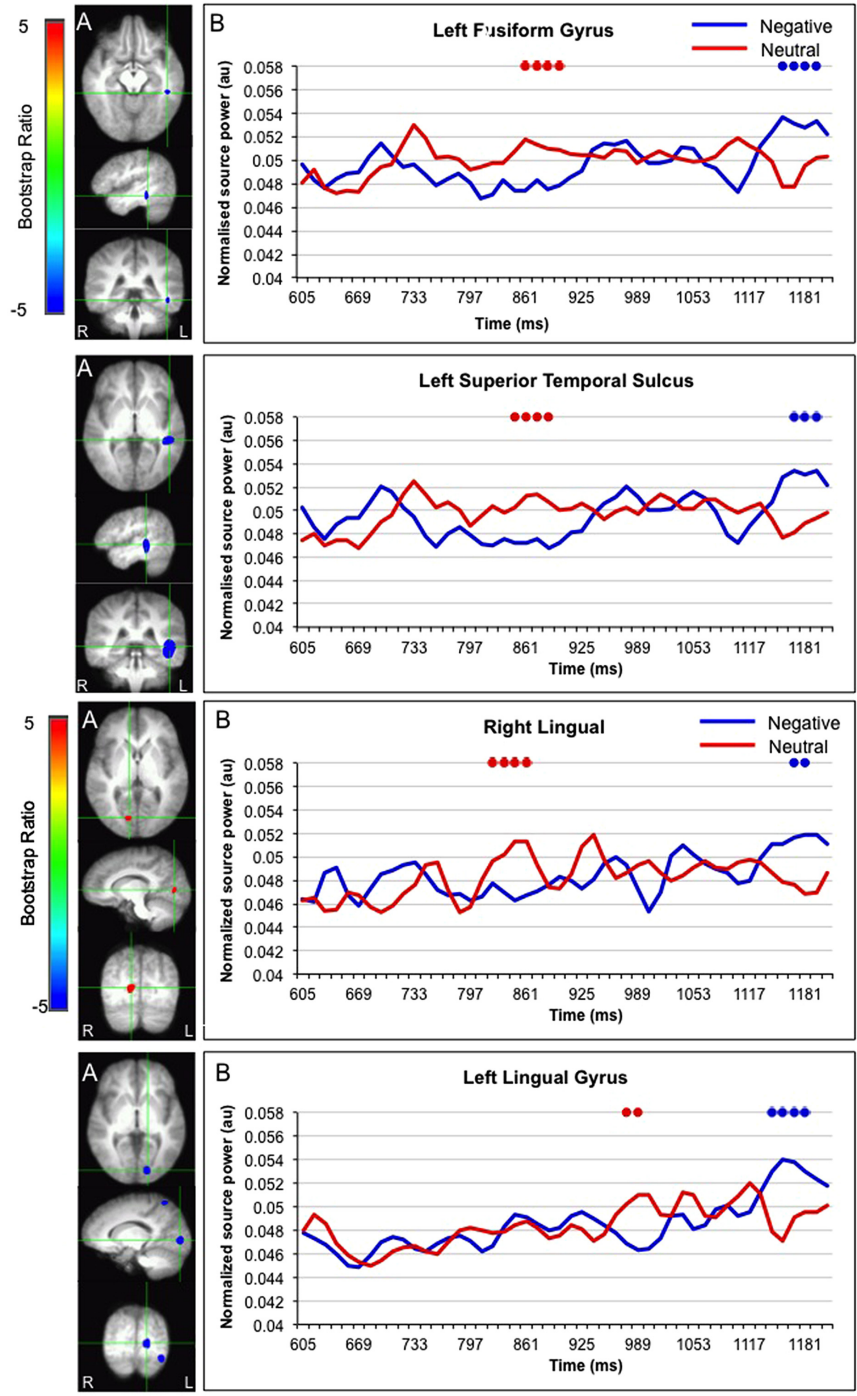
**Sources initially showing stronger activation for faces paired with neutral as compared to negative sentences, and subsequently stronger activation for faces paired with negative as compared to neutral sentences. (A)** The brain areas associated with the PLS bootstrap ratio of less than –3 and more than 3 in the LV1. **(B)** Corresponding ER-SAM waveforms from sources identified in PLS bootstrap ratio maps. Blue dots denote bootstrap ratios <–3, and red dots denote bootstrap ratios >3. Since no significant differences were found for 0–600 ms, the x-axis begin at around 600 ms in order to fully visualize the emotion-modulated differences observed.

Interestingly, activation in the fusiform gyrus, left superior temporal gyrus, and bilateral lingual gyrus initially showed a larger response for faces paired with neutral versus negative sentences, but ultimately showed a larger response for faces paired with negative sentences (**Figure 5**).

In summary, presentation of negative versus neutral information led to differences in the subsequent viewing and processing of neutral faces.

### EYE MOVEMENT-LOCKED MEG ANALYSIS

Given that differences in viewing neutral faces following negative versus neutral sentences were not observed until *after* participants entered the eye region of the face (∼400 ms after stimulus onset), we also examined temporal differences in neural activity for the above regions that were time-locked to the moment when eye movements first entered the eye region of the face, i.e., this was marked as time 0. ER-SAM waveforms were extracted for each participant and condition (0–1000 ms), and source activity was averaged within time bins of 100 ms and emotion-modulated differences were examined using paired sample *t*-tests. After correcting for multiple comparisons, the only significant time period to emerge between Face2-Negative and Face2-Neutral was 700–800 ms in the precuneus [*t*(11) = 4.19, *p* < 0.001]. Specifically, following fixation of the eye region, activity in the precuneus was stronger for faces presented after a negative versus neutral sentence.

## DISCUSSION

The presentation of negative information changed the way in which neutral faces were subsequently viewed and the neural systems that were engaged following early perceptual processing. Further, the novel use of simultaneous eye movement and MEG recordings allowed us to explore the direct relationship between the influence of emotion on viewing behavior and neural activity. This was achieved by locking the MEG signal to specific eye movement behavior; here, when the eye movements first moved into the eye region of the neutral faces. In the next sections, we discuss our results in light of prior findings regarding how emotion may modulate processing of neutral information, and how the current work may inform theories regarding the influence of emotion on perception and memory.

### EMOTION-MODULATED VIEWING OF NEUTRAL FACES

Emotional details influenced the immediate viewing to neutral faces. Specifically, when faces were presented after a negative versus neutral sentences, there was increased viewing to the eye region of faces during the first gaze to that region. While the first gaze occurred between 400 and 1400 ms after the onset of Face 2, it was the very first instance that the eye region had been fixated. Given that direct fixation allows more information to be derived from the fovea than could otherwise be gleaned from the periphery, this may suggest that perceptual processing of the eyes themselves is still occurring at this point (even at 400 ms post-onset). Therefore this change in duration of viewing may reflect a change in the manner by which perceptual processing is occurring (Aviezer et al., 2008). Previous studies have shown that the viewing of threat-related versus non-threat-related facial expressions is also characterized by increased viewing to the eye region (Adolphs et al., 2005; Gamer and Buchel, 2009; Itier and Batty, 2009). Within the current experiment, the neutral faces may have taken on emotional qualities and elicited viewing patterns similar to those reported for the viewing of faces that are actually expressing emotion. Further work is needed to directly compare viewing of faces expressing emotion and viewing of neutral faces presented with emotional details.

Alternatively, emotion-modulated differences in viewing neutral faces may reflect processing differences that occur after perceptual processing has been completed. Specifically, after building a visual percept of the neutral face (Face 1), participants may have bound the sentence information to the neutral face during subsequent viewing (Face 2). Faces paired with negative versus neutral sentences may have invoked a greater need to appraise or reappraise the face, leading to increased viewing of regions of the face that are most informative (i.e., the eyes). Consistent with the axiom that the eyes may be a “window to the soul,” previous research shows that viewing to the eye region is associated with the assessment of interest, threat, and intentions of other people (Haxby et al., 2002; Spezio et al., 2007).

### EMOTION-MODULATED PROCESSING OF NEUTRAL FACES

In addition to examining emotion-modulated differences in viewing, we also examined the extent to which presenting negative information immediately prior may influence underlying neural processing of the neutral face, and precisely when emotion may exert its influence. Specifically, if emotion modulated processing of neutral faces occurred early, i.e., within the first 250 ms, then this would suggest that emotion changes perceptual processing of subsequent information. However, our analysis did not reveal any significant spatiotemporal differences in processing neutral faces presented after negative versus neutral sentences within the first 250 ms after stimulus onset. This is consistent with the MEG study by Morel et al. (2012), which also did not find any early neural differences between faces paired with negative versus neutral information. While Morel and colleagues only examined neural differences in the first 0–200 ms after stimulus onset, the current results showed that emotion-modulated neural differences emerged 600–1200 ms after stimulus onset. This time frame is consistent with, albeit a little later than the EMM results which revealed emotion-modulated viewing differences between 400 and 1400 ms.

The current pattern of results suggests that emotion may modulate later stages of processing such as binding the sentence to the face and/or the appraisal of the neutral face (at least immediately during encoding). The current results also suggest that the presentation of emotional information may drive participants to direct more viewing to informative regions of the face such as the eyes. In doing so, this may increase neural activity in regions implicated in emotion processing, resulting in the construction of a different type of internal representation as compared to faces paired with a neutral sentence. Interestingly, whereas the current study found that emotional information modulated the immediate processing of neutral faces between 600 and 1200 ms, Smith et al. (2004) found that emotion modulated subsequent retrieval of neutral items as early as 300 ms following stimulus onset. This suggests that not only does association with emotion influence one’s representation of neutral information, but that this may become stronger over time (resulting in faster retrieval) as the links between emotional and neutral items become consolidated or as the emotional information becomes a feature of (i.e., blended into) the neutral item.

In support of the notion that emotion may lead to the construction of a different type of representation, processing neutral faces following negative versus neutral sentences elicited stronger activity in neural regions implicated in emotion processing such as the amygdala, cingulate, and medial prefrontal cortex (Lane et al., 1997; Culham and Kanwisher, 2001; Anderson et al., 2003; Kensinger and Corkin, 2003; Adolphs et al., 2005). It is possible that increased activity in these regions drove subsequent changes in the posterior cortices, leading to increased appraisal or reappraisal of neutral faces following negative as compared to neutral sentences. Consistent with this, activation differences in emotion-related regions were followed by differences in neural activity in the precuneus and inferior parietal lobule, which have been linked to the maintenance of attention and the processing of salient information (Culham and Kanwisher, 2001; Singh-Curry and Husain, 2009). In the current results, activation peaks in these two parietal regions were followed by peaks in neural regions in other emotion processing regions such as the cingulate and medial prefrontal cortex (Culham and Kanwisher, 2001; Kensinger and Corkin, 2003). This may reflect the retrieval and/or attachment of emotional information to a neutral face (Maratos et al., 2001; Smith et al., 2004; Fenker et al., 2005; Medford et al., 2005) and/or the appraisal and assessment of the neutral face in light of the negative sentence.

When we explored the precise relationship between eye movement behavior and neural activity further by locking the MEG results to the time at which participants’ eye movements first moved into the eye region of the neutral faces, activity in the precuneus was observed. Specifically, presentation of neutral faces following negative versus neutral sentences elicited increased activity in the precuneus 700–800 ms following fixation on the eye region (∼1100–1200 after stimulus onset). In addition to attention, the precuneus has also been implicated in episodic memory retrieval, mental imagery, self-referential activity, and theory of mind (ToM); more specifically, relative increases in responses arise in the precuneus when making judgments of others’ actions and intentions compared to making judgments of the self (Cavanna and Trimble, 2006). It has been suggested that judgment of others may elicit a particularly vivid representation of the self in order to “put oneself in another’s shoe”. Thus, the viewing of neutral faces following emotional versus neutral information may increase the likelihood of retrieving the specific information presented before the face, engaging in mental imagery (i.e., some of the sentences were quite graphic), and/or evaluating the face with regards to its potential congruence with the sentence, risk, and/or effect on the self.

A number of regions, namely the fusiform gyrus, STS and bilateral lingual gyrus, initially showed stronger responses for faces presented after neutral versus negative sentences, but then showed stronger responses for faces presented after negative versus neutral sentences. The STS has been shown to be involved in inferring the intentions and attributes of other people (Winston et al., 2002) and the representation of biographical information (Haxby et al., 2002; Todorov et al., 2007). In this way, emotional information may modulate and change encoding processes, possibly via enhanced activation of visual processing regions in the occipital cortex and specific face processing modules in the fusiform gyrus (Halgren et al., 2000; Prince et al., 2009). Critically, these processes may be delayed for neutral faces presented after negative versus neutral sentences because the emotional significance of the face and/or the congruence between the physical properties of the face and preceding biographical information must first be evaluated or considered. Taken together, results from MEG suggest that emotional information may influence the processing of neutral faces via a parietal-limbic-frontal network that may drive, as well as be modulated by, eye movement behavior.

Interestingly, processing of faces presented after neutral versus negative sentences elicited stronger activation in the superior frontal gyrus and within the medial temporal lobe, i.e., in the parahippocampal gyrus, which may reflect enhanced processing of the sentence and the blending/binding of that information to the neutral face. This was somewhat counterintuitive as it seemed that it would be more important and relevant to bind emotionally salient information to the neutral face rather than affectively neutral information. However, there has been some suggestion in the literature showing that while processing and memory of associated neutral information is mediated by the medial temporal lobes, processing and memory of associated emotional information may be more dependent on regions such as the amygdala and temporal poles (Phelps and Sharot, 2008). For example, Todorov and Olson (2008) presented participants with neutral faces paired with positive or negative sentences and later asked them to rate each face on scales of likeability, trustworthiness, and competence, and to make a force-choice judgment of preference. They found that while healthy controls and patients with hippocampal damage showed learning effects (i.e., preferring faces previously paired with positive versus negative behaviors), patients with damage to the amygdala and temporal poles did not. In light of this, it is possible that in the present experiment, participants built different types of representations for the face-sentence pairings depending on whether the sentence was negative or neutral. Specifically, participants may have built a parahippocampal-based representation of faces with neutral sentences, and an emotion system-based representation of faces with negative sentences. However, it is important to note that in the current study, differences in neural activity were relative rather than absolute. In this way, the evidence lends support to the notion that binding/blending of emotional versus neutral information may depend more on one system versus another, not that they rely only on one system versus another.

### CONSIDERATIONS AND FUTURE DIRECTIONS

There are a few considerations regarding the current study that should be noted. First, we preprocessed the MEG data using a PCA approach which is widely accepted and commonly used in EEG and MEG studies to identify and reject potential large artifacts such as those related to eye blinks and movement. However, small residuals below our cutoff threshold of 1.5 pT, such as those related to saccades, may remain in the data, and affect the quality of source localization, especially in anterior frontal regions. Previous studies have shown that eye artifacts can ‘leak’ into anterior frontal, inferior temporal, and occipital sources (Bardouille et al., 2006; Carl et al., 2012) and/or mask weaker activity from these regions (e.g., Fatima et al., 2013). This is an inherent problem in MEG studies and various methods have been applied to reduce artifact noise, such as the PCA approach used in the present study, manual and automated rejection of trials containing artifacts (e.g., de Hoyos et al., 2014; Gonzalez-Moreno et al., 2014), dipole modeling (e.g., Berg and Scherg, 1991), and independent component analysis (ICA; e.g., Mantini et al., 2008; Fatima et al., 2013). To the best of our knowledge, no study has systematically reviewed the above mentioned methods and compared their relative efficacy. However, no method is likely perfect, and MEG results need to be interpreted in the context of converging evidence. This becomes especially relevant in studies where emotional stimuli are used (e.g., faces expressing emotion and IAPS pictures) and differences in eye movements are elicited – even if this is not explicitly measured. One solution is to instruct participants to maintain a central fixation and not move their eyes. However, while this reduces potential eye movement artifacts, it also reduces ecological validity and results in different cognitive process (e.g., inhibition of eye movements) as compared to when participants are allowed to freely explore. In the present study, it is important to note that although differences in eye movements were found between the experimental conditions, it is unlikely that they were time-locked to the presentation of the face (i.e., scanning each face in exactly the same manner). Further, the neural differences that we observed were consistent with those reported in other emotion and face processing studies, and not limited to anterior regions near the eyes. Therefore, differences in time-locked neural activity likely reflect underlying cognitive processes, and not merely differences in scanning.

Second, in the present study, the delay between the sentence and second presentation of the face was short (500 ms). With such a short time window, one could argue that differences in viewing Face 2 represent a carryover effect from processing the preceding sentence, or that there are stimulus-general processing effects that occur in response to the presentation of the emotional information. However, if this were the case, then presumably emotion-modulated differences should have occurred very early during Face 2 and dissipated over time. That is, it is not clear why either a carryover effect or a stimulus-general effect of emotion would be initially oﬄine and then re-invoked later during processing. The fact that neural differences were observed later during viewing suggests that it may be the retrieval and/or conceptual evaluation of the face that is influencing ongoing processing, rather than a change in the general state. Although we did not observe any differences in early time windows, it will be important for future studies to control for potential carryover effects.

Lastly, this study was limited to the examination of the influence of negative emotions on the immediate processing of subsequent neutral information. It will be important for future work to explore how valence affects processing of neutral information (i.e., positive versus negative), and how emotions may alter long-term memory of neutral information (e.g., Mather and Knight, 2008; Guillet and Arndt, 2009; Riggs et al., 2010; Madan et al., 2012; Sakaki et al., 2014).

## CONCLUSION

This work demonstrates that emotions can alter processing of otherwise neutral information by changing overt viewing patterns. This adds to the considerable literature showing that visual processing is determined not only by bottom–up physical characteristics, but also by top–down influences such as prior knowledge, memory, and context (Aviezer et al., 2008; Ryan et al., 2008). Processing of faces paired with negative sentences relied more on a neural network mediated by regions involved in emotional processing, whereas processing of faces paired with neutral sentences relied more on regions involved in memory (i.e., the parahippocampal gyrus). This suggests that not only do emotional details influence subsequent online processing of neutral information to which it pertains, but it may also alter the type of representation that is formed. This may have important implications for understanding clinical disorders such as anxiety, post-traumatic stress disorder, and depression where seemingly neutral stimuli may trigger an emotional response. Further, while Herdman and Ryan (2007) demonstrated the feasibility of combining eye movement and neuroimaging data within a single paradigm, the current study represents the first application of this approach to the study of emotion and memory. More generally, the simultaneous recording of eye movements and neural activity can serve to elucidate the relationship between behavior, neural activity, and cognitive processing.

## Conflict of Interest Statement

The authors declare that the research was conducted in the absence of any commercial or financial relationships that could be construed as a potential conflict of interest.

## Footnotes

1 Of note, although emotion includes both positive and negative valenced information, which may have different consequences when paired with neutral information (e.g., Morel et al., 2012), we focused on the effects of negative emotion. For simplicity, all references to ‘emotion’ in this paper concerns negative emotion only.
